# Enhancement of tumor initiation and expression of KCNMA1, MORF4L2 and ASPM genes in the adenocarcinoma of lung xenograft after vorinostat treatment

**DOI:** 10.18632/oncotarget.3536

**Published:** 2015-03-12

**Authors:** Wei-Ying Kuo, Chun-Yi Wu, Luen Hwu, Jhih-Shian Lee, Cheng-Han Tsai, Kang-Ping Lin, Hsin-Ell Wang, Teh-Ying Chou, Chun-Ming Tsai, Juri Gelovani, Ren-Shyan Liu

**Affiliations:** ^1^ Department of Biomedical Imaging and Radiological Sciences, National Yang-Ming University, Taipei, Taiwan; ^2^ Department of Biomedical Imaging and Radiological Science, China Medical University, Taichung, Taiwan; ^3^ Molecular and Genetic Imaging Core/Taiwan Mouse Clinic, National Comprehensive Mouse Phenotyping and Drug Testing Center, Taipei, Taiwan; ^4^ Department of Electrical Engineering, Chung Yuan Christian University, Chungli, Taiwan; ^5^ Holistic Medical Device Development Center, Chung Yuan Christian University, Chungli, Taiwan; ^6^ Institute of Clinical Medicine, National Yang-Ming University, Taipei, Taiwan; ^7^ Department of Medicine, National Yang-Ming University, Taipei, Taiwan; ^8^ Department of Biomedical Engineering and Karmanos Cancer Institute, Wayne State University, Detroit, MI, USA; ^9^ National PET/Cyclotron Center and Department of Nuclear Medicine, Taipei Veterans General Hospital, Taipei, Taiwan

**Keywords:** Lung cancer, ALDH activity, cancer stem cells, suberoylanilide hydroxamic acid (SAHA)

## Abstract

Cancer stem cells (CSCs) are usually tolerant to chemotherapy and radiotherapy and associated with tumor relapse. Suberoylanilide hydroxamic acid (SAHA), a histone deacetylase inhibitor (HDACI), is currently being used in clinical trials of lung cancer. However, SAHA facilitates the formation of induced pluripotent stem cells from somatic cells. We hypothesized that SAHA would mediate the CSCs properties and subsequently confer a more malignant phenotype in lung cancer. Transfected H1299 lung cancer cells, which stably expresses a triple fused reporter gene (DsRedm-Fluc-tTKsr39) under the control of CMV promoter was used to establish a xenograft mouse model. After the treatment of SAHA, H1299 cell line and tumor xenografts were sorted by fluorescence-activated cell sorting (FACS) based on aldehyde dehydrogenase (ALDH) activity. We found that SAHA could suppress the growth of xenografted H1299 tumors with decreased proportion of ALDH^br^ lung cancer cells indicating that SAHA may target CSCs. However, SAHA significantly enhanced the tumor initiating capacity and the expression of malignant genes such as KCNMA1, MORF4L2 and ASPM in the remaining living ALDH^br^ cells. These findings suggested that SAHA treatment created a more drug-resistant state in residual ALDH^br^ cells. The *in vivo* imaging technique may facilitate searching and characterization of CSCs.

## INTRODUCTION

In the cancer stem cell (CSC) paradigm, cancer originates from the uncommon cells with characteristics of pluripotency and self-renewal [1]. CSCs were first identified in leukemia and more recently in solid tumors. This subpopulation of cancer cells has ability to escape chemotherapy and driving tumor recurrence [2]. It is urging to develop effective therapeutic approaches to eliminate CSCs. However, a long-standing problem is the paucity of specific markers to identify and isolate CSCs from common tumor cells and to investigate their roles in tumorigenesis. Previous data showed that tumorspheres, CSCs-like clusters, can be generated *in vitro* by non-adherent suspension culture in serum-free medium, and they have been widely used to study underlying key molecular pathways [3]. Mounting evidences suggest that the tumor microenvironment is responsible for conditioning the stem cell status itself. The *in vitro* system has been questioned because of the entire differences between *in vitro* and *in vivo* systems in microenvironment. Side population (SP) technique and flow cytometry using cell surface markers have been applied to isolate CSC, but the specificity of these two methods is under debate. Previous studies reported that non-SP cells and CD133^─^ cells can also generate tumors in NOD/SCID mice [4, 5].

Regarding the limitations in the isolation procedures, especially those used stem cell surface markers, would result in CSC “injury”, we designed an *in vivo* method using intracellular markers of stem cells which were identified in various human cancers to isolate CSCs from xenograft tumors in animal model. Aldehyde dehydrogenases (ALDHs) are detoxifying enzymes within a superfamily. In fact, the expression level of ALDH in stem cells usually high enough to protect them against oxidative insult, suggesting their well-known longevity. ALDH converts retinol to retinoic acid, a modulator of cell and stem cell proliferation. Elevation of the level of ALDH activity has been seen in stem cell populations of breast cancer [6], lung cancer [7], liver cancer [8] and colon cancer [9]. An ALDEFLUOR kit (Stem Cell Technologies) designed for precise identification and isolation of ALDH-bright CSCs using specific inhibitor for ALDH activity─ diethylaminobenzaldefyde (DEAB) was thus applied in this study.

Histone deacetylase inhibitors (HDACIs) can induce hyperacetylation of specific proteins, recently considered as a new solution to inhibit cell proliferation and promote differentiation of various hematologic and solid tumors [10, 11]. Suberoylanilide hydroxamic acid (SAHA, Vorinostat), an HDACI, was approved by FDA for treatment of cutaneous T-cell lymphoma in 2004 [12]. Recent investigations demonstrated that SAHA treatment can suppress the expression of the stem cell marker CD133 in glioma [13]. In addition, SAHA can also inhibit the ability to proliferation, self-renewal, migration, and invasion in human pancreatic CSCs by up-regulation of miR-34a [14]. These results implied that SAHA could be a potential agent for the therapy against CSCs. However, some studies revealed that SAHA leads to the increase of the stem cell markers in epithelial–mesenchymal transitions (EMT) phenotypic prostate cancer cells [15, 16]. These findings are in consistent with the clinical results of HDACIs, which have shown promise efficacy in hematological malignancies while disappointed effects in epithelial cell-derived cancers. The detailed mechanism of this phenomenon remains to be elucidated.

In the current study, we aim to determine the significance role of SAHA in the mediation of CSCs in lung cancer. The ALDEFLUOR assay and FACS analysis were used to isolate CSCs from human lung carcinoma grown as xenografts on nude mice. The results showed that SAHA retards the growth of H1299 xenografts and decreases CSC population, but induces EMT phenotype and activates pluripotency associated program in the residual CSCs. Our results provide a possible mechanism for the limited treatment response of HDACIs in the clinical trials on the epithelial cell-derived cancer.

## RESULTS

### SAHA enhances the expression of CSC characteristics *in vitro*

**Figure 1:**
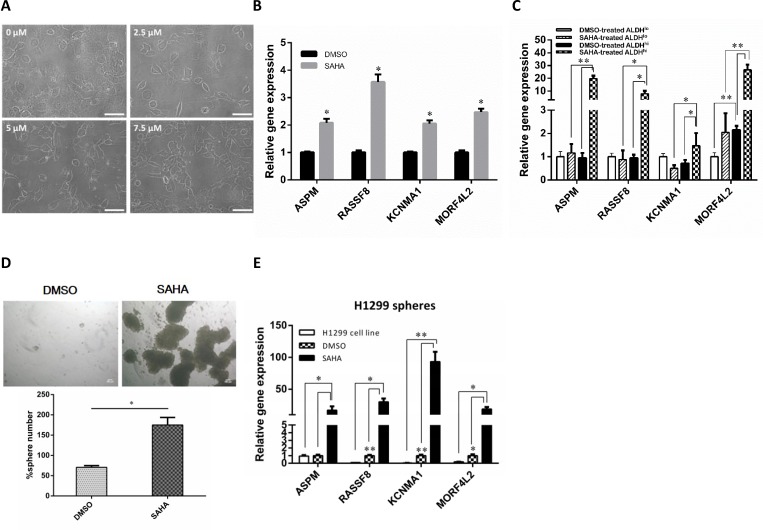
SAHA enhances the CSC characteristics *in vitro* H1299 cells were incubated in the medium containing various concentration of SAHA for 72 h. (A) Morphological examinations were performed. Scale bars, 100 μm. (B) qRT-PCR of H1299 cells treated with SAHA or vehicle (DMSO). The results showed that the relative mRNA expression of ASPM, KCNMA1, RASSF8 and MORF4L2 increased following treatment when compared with DMSO control (the value of control was designed as 1, *, *p*<0.001). (C) qRT-PCR analysis in sorted H1299 cells confirmed the elevated expression of metastases-, stemness- and drug resistance-related genes in ALDH^br^ cells after SAHA treatment. *, *p*<0.05; **, *p*<0.001. (D) Representative images (top) and quantification (bottom) of spheroid formation in H1299 cells treated with SAHA. Scale bars, 100 μm. Data represent means ± s.e.m. (n=3). Asterisk indicates *p*<0.001, compared with control cells (Student's *t*-test). (E) SAHA increased the expression of gene associated with stemness, metastasis and drug resistance in tumorspheres. qRT-PCR was conducted with parental cells and sphere-forming cells for stem cell and EMT-related genes. Delta-delta-CT was calculated, considering β-actin as internal control. Three experiments were run in triplicates. *, *p*<0.05; **, *p*<0.001.

To investigate the effects of SAHA on cell morphology, we treated H1299 cells with SAHA, and found that H1299 cells treated with SAHA caused elongation and dissemination of fibroblastoid morphology in a dose-dependent manner. In contrast, H1299 cells treated with DMSO displayed a cobblestone appearance, a typical morphology of epithelial cells (Fig. 1A). These results suggest that treatment of lung cancer cell with SAHA leads to the induction of EMT phenotype. Previous studies have shown that HDACIs could induce EMT in tumor cells [15, 16], and the cells with EMT phenotype have been demonstrated to be the source of cancer stem-like cells [17-19]. To address this issue, we assessed the expression of genes associated with metastatic ability and stem cell function such as KCNMA1 and ASPM, which has been reported earlier to serve as prognostic molecular markers of the metastatic process and cancer stem cell maintenance, respectively [20, 21]. The results from quantitative reverse transcription PCR (qRT-PCR) showed that the treatment with SAHA led to increased expression of genes involving in stemness (ASPM, stem cell programs), metastasis (KCNMA1, RASSF8), and drug resistance (MORF4L2) (Fig.1B). We further investigated the effect of SAHA on CSC populations by using qRT-PCR and found that all the analyzed genes was strongly up-regulated by at least 10-fold in SAHA-treated ALDH^br^ cells when compared with untreated cells (Fig.1C).

The capacity of self-renewal is one of the features of CSCs. To further verify the impact of SAHA on cancer stem cell properties, we performed the sphere formation assay as previous described [6]. The result showed that cells treated with SAHA formed more spheres than untreated cells did (Fig.1D). We next examined if SAHA could enhance the expression of stem cell-associated genes in tumorspheres. As shown in Fig. 1E, the levels of all gene sets were increased in sphere cells when compared with the controls. As expected, these genes were significantly enriched in sphere cells after SAHA application. Overall, these findings confirm that treatment of lung cancer cells with SAHA leads to the enhancement of CSC characteristics, which correlates with EMT phenotype.

### Efficacy of SAHA treatment in the H1299 xenograft model

**Figure 2:**
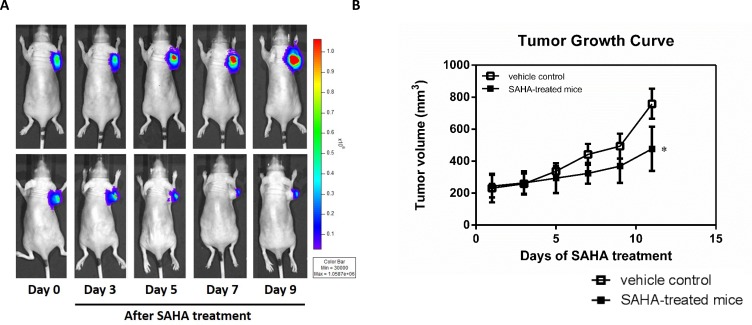
SAHA effectively inhibits the growth of H1299 tumor cells (A) Bioluminescent images of mice bearing H1299-CMV-Luc tumors before and after SAHA treatment. (B) Tumor growth curve of the subcutaneous H1299-CMV-Luc lung cancer xenograft in mice daily treated with vehicle alone or SAHA (100 mg/kg, i.p.). Data are presented as the mean tumor volume ± S.E. of the surviving animals in each groups. All groups contained five mice.

To monitor the tumor growth and trace the tumor cells *in vivo*, we labelled H1299 cells with a luciferase reporter system. The activity of SAHA against the growth of lung cancer cells *in vivo* was examined in the H1299 human non-small cell lung cancer xenograft, which was inoculated subcutaneously in nude mice. The tumor progression rate was assessed by *in vivo* luciferase bioluminescent imaging. In the treatment group, the signals in the tumor are significant lower than that in vehicle-treated control tumor (Fig. 2A). Daily administration of SAHA with the dosage of 100 mg/kg/day caused significant suppression of the growth of established H1299 tumors; reduction of 63% tumor volume compared with that of the vehicle-treated control animals (Fig. 2B). Each animal receiving 100 mg/kg/day SAHA survived for at least 10 days. These results indicate that SAHA effectively reduces the tumor growth of H1299 xenografts *in vivo* at the dose of 100 mg/kg/day.

### Expression of cancer stem cell markers and EMT markers in fresh xenograft

**Figure 3:**
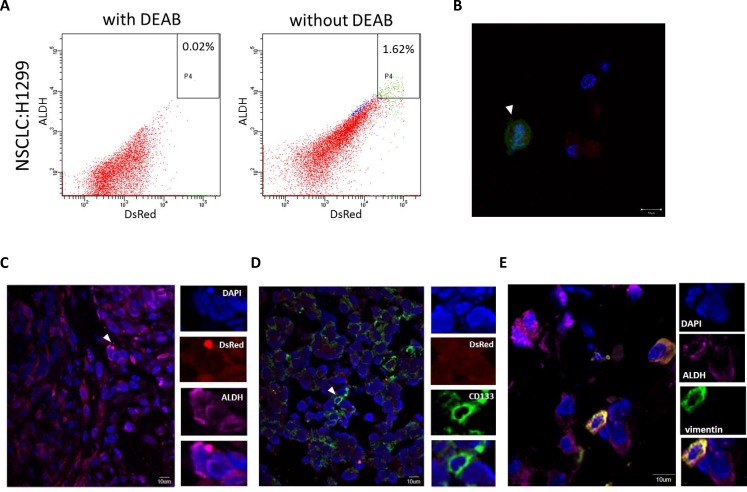
Cancer stem cells and EMT markers were detected in xenografted lung tumor cells (A) Fresh H1299 xenograft tumors were directly separated for non-CSC (ALDH^lo^ cells) and CSC (ALDH^br^ cells) populations by the ALDEFLUOR assay (right panel). Baseline fluorescence was established by inhibiting ALDH activity with DEAB (left panel) and used to generate a gate to identify ALDH^br^ cells, revealing a subpopulation (~2%) of ALDH^br^ tumor cells. (B) Confocal microscopy imaging indicated the existence of ALDH^br^ cells (green, arrow) with nuclei identified by DAPI (blue areas). (C, D and E) Immunofluorescent staining shows ALDH, CD133 and vimentin expression in H1299 xenograft tumor tissues. Abbreviations: ALDH, aldehyde dehydrogenase; DEAB, diethylaminobenzaldehye.

ALDH activity has been applied for identifying CSCs in a variety of tumor types [6, 7, 9]. We used the ALDEFLUOR assay to quantitate the percentage of ALDH^br^ cells in xenografted lung tumors (Fig. 3A). In each experiment, the samples were stained simultaneously with Aldefluor and DEAB for identifying the ALDH^br^ population. A subpopulation of ALDH^br^ cells was detected by flow cytometry (1.62% ±0.02% of the total cell population) and was also observed by fluorescent microscopy (Fig. 3B).

CD133 and ALDH have been applied to define CSCs in multiple epithelial cancer [8] including lung cancer [22]. We examined the expression of these markers in mouse xenografted tumors. Immunofluorescence staining confirmed the expression of ALDH and CD133 in fresh xenograft tissues (Fig. 3C and 3D). The process of EMT has been played a critical role in tissue and vessel invasion. Regarding that CSCs also express some features of EMT, we tried to determine whether ALDH^br^ tumor cells harbored EMT phenotype such as expression of the EMT markers. To test this phenomenon, we co-stained with a fluorescent dye-conjugated antibody for the EMT marker vimentin. Immunofluorescence staining confirmed that ALDH^br^ cells co-expressed vimentin in fresh xenografted lung cancer tissues (Fig. 3E), indicating the fresh lung cancer cells express multiple potential cancer stem cell markers and some of these cells also possess EMT properties.

### SAHA reduces the population of ALDH^br^ cells in H1299 xenografts

**Figure 4:**
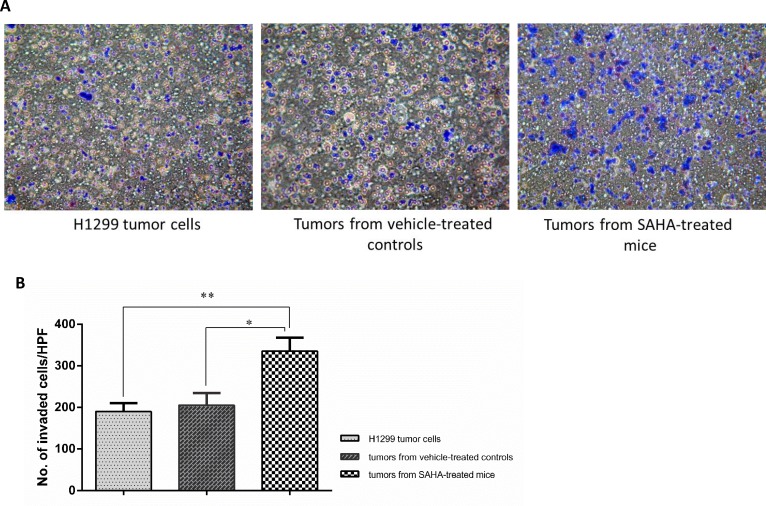
Continuous administration of SAHA increased the invasion ability in ALDH^br^ cells Phase-contrast microscopy images (top) and invasion ability (bottom) of ALDH^br^ cells from H1299 lines, SAHA-treated xenografts or vehicle-treated controls. Data represent mean ± SD (n=3). Asterisks indicate *P*< 0.001, compared with H1299 cell line (Student's *t*-test).

To further validate the antitumor effects of SAHA *in vivo*, we isolated ALDH^br^ cells from xenograft tumors based on the previously described [23-29]. We also performed ALDEFLUOR assay to isolate ALDH^br^ cells and found that the percentage of ALDH^br^ cells derived from H1299 xenografts of mice treated with SAHA was 0.2% ± 0.04 (Fig. 4A), which was around a 11-fold reduction (Fig. 4B) compared with that of the cells directly isolated from the mice treated with vehicle alone (Fig. 4A). Confocal microscopic images further confirmed the existence of ALDH^br^ cells in our lung cancer xenograft models and effective suppression of the ALDH^br^ cells by SAHA treatment in the *in vivo* experiment (Supplement 2.).

### SAHA enhances the migration ability in ALDH^br^ cells sorted from H1299 xenografts

**Figure 5:**
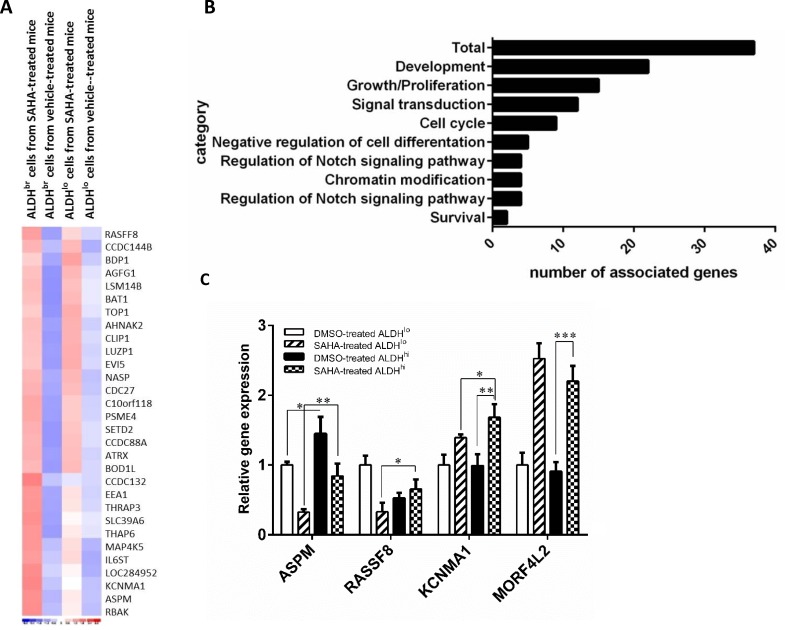
Functional effects of SAHA in ALDH^br^ cells from mouse xenografts Profiling of ALDH^br^ cells from SAHA-treated mouse xenografts. (A) Supervised hierarchical clustering of 37 differentially expressed transcripts between ALDH^br^ cells from mouse xenografts treated with or without SAHA. Colored spots indicate upregulated (red) or downregulated (blue) genes from microarray analysis. (B) Gene ontology of RNA Microarrays show that many genes involved in “development”, “cell cycle” and “chromatin modification” are affected due to SAHA treatment. (C) Quantitative reverse transcription-PCR (qRT-PCR) validation of differentially expressed genes. cDNAs of ALDH^br^ and ALDH^lo^ subpopulations were obtained from mouse xenografts treated with SAHA or vehicle controls. Expression levels were normalized to GAPDH. Data represent mean ± SEM (n=3).

To determine the metastatic capacity of these cell populations after SAHA treatment, tumor cells from mouse xenografts were sorted using ALDEFLUOR assay and the ALDH^br^ cells from xenografts of the mice treated with SAHA or vehicle alone were further examined by Matrigel invasion assay. As shown in Fig. 5, the percentage of ALDH^br^ H1299 cells from the mice treated with SAHA capable of invasion through Matrigel was at least 1.9-fold higher than that of the Aldefluor-positive H1299 cells from vehicle-treated controls (*P*< 0.05). These results suggest that although SAHA suppressed the growth of tumor xenografts and decreased the CSCs population *in vivo*, the residual CSCs seems more invasive.

### Profiling of ALDH^br^ subpopulation from the xenografts of mice treated with SAHA identifies the upregulation of CSC-associated genes

One novelty of this study was to identify the potential target against CSCs by *in vivo* molecular imaging method. To understand the biology of ALDH^br^ cells from mice xenografts treated with SAHA, we analyzed the gene profiles of ALDH^br^ cells using microarray technique. Unsupervised hierarchical cluster analysis was applied to the four samples (ALDH^br^ and ALDH^lo^ cells from H1299 xenografts with SAHA administration or vehicle controls) and found 37 interested genes with different expression between ALDH^br^ cells and SAHA-treated mouse xenografts and from vehicle-treated controls (Fig. 5A, fold change > 2.0, *P*< 0.05). Owing to the observed reduction of the ALDH^br^ population, we expected that the downregulation effect should be noticed in these CSC-related pathways after HDAC inhibitor treatment. However, these gene set (ALDH3A2, CDC42, FGF7, stem cell programs) were not downregulated instead of more pronounced and highly upreglated after SAHA treatment. Based on previous literatures, the rest of upregulated genes were associated with stem cell functions, including cell cycle regulation, mitosis, and proliferation or biological processes, including transporters, protein binding, regulation of transcription, signal transduction, metabolism and cell movement (Fig. 5B).

Gene expression of four upregulated genes in ALDH^br^ cells from SAHA-treated mouse xenografts was valiadated by qRT-PCR, It showed significantly higher expression of the pluripotency genes, ASPM and RASSF8, at the mRNA levels compared to the ALDH^br^ cells from vehicle-treated mouse xenografts and unsorted population. It also showed higher mRNA expression of the EMT gene (KCNMA1) and drug-resistant related gene, (MORF4L2) (Fig. 5C). These results illustrate that the genes associated with pluripotency are induced by SAHA and help to unravel the mechanism of HDAC inhibitor on proliferation and survival of human lung CSCs.

### Enhanced tumorigenicity of SAHA-treated ALDH^br^ lung cancer cells

**Figure 6:**
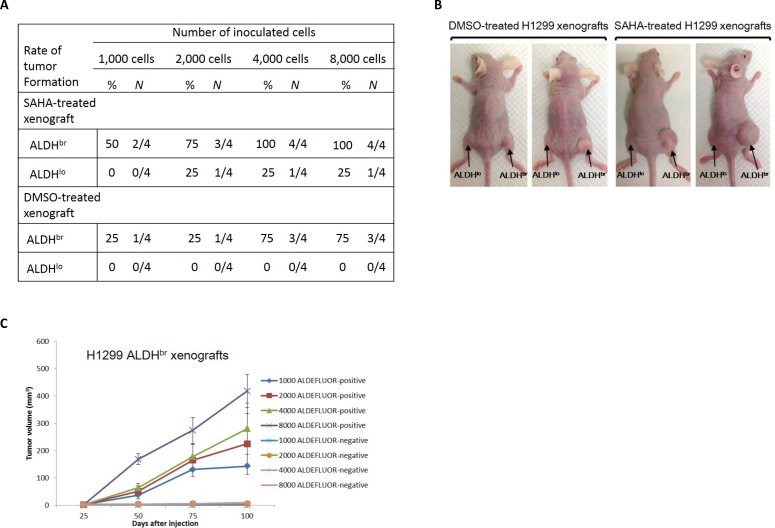
The ALDH^br^ cell population from SAHA-treated H1299 xenografts displays enhanced properties of CSCs (A) Incidence of tumor formation from isolated ALDH^br^ lung cancer cells. For each group, varying numbers of ALDH^br^ and ALDH^lo^ cells were injected and tumor growth was measured over a 100-d interval. (B) Representative photographs of SAHA or vehicle-treated xenografts derived from respective ALDH populations after a 3-months tumor development. The size of xenograft derived from ALDH^br^ cells was significantly larger than that derived from ALDH^lo^ cells in both groups. (C) Tumorigenicity assay. The ALDH^br^ cell population from SAHA-treated H1299 xenografts was capable of regenerating phenotypic heterogeneity of the initial tumor after passage in nude mice. No tumor was detected when 8,000 of ALDH^lo^ cells were injected, whereas ALDH^br^ cells produced tumors that grew at a rate that directly correlated with the number of cells injected.

To further determine the efficacy of SAHA treatment on cancer stem cells sorted from xenografts, we electronically sorted ALDH^br^ and ALDH^lo^ cells from SAHA or vehicle-treated H1299 xenografts, respectively. Variable numbers of sorted cells were immediately implanted into nude mice by subcutaneous injection, and tumor formation was observed up to 24 weeks to avoid underestimating the tumor initiating cells (TIC) frequency by a short observation time. The capacity of *in vivo* tumor formation with sorted cells was different between these groups. Only the injection of ALDH^br^ cells gave rise to visible tumors, particularly at low numbers of injected cells (1,000 cells), whereas little or no tumors were observed with ALDH^lo^ cells, even when as many as 8,000 cells were injected (Fig. 6A). Moreover, we found that the tumor volume and incidence of tumor formation by ALDH^br^ tumor cells from mice received SAHA treatment were larger and higher than that of the mice received DMSO treatment (Fig. 6B). As indicated in Fig. 6C, the size and latency of tumor formation correlated with the number of injected cells, where 1,000 ALDH^br^ cells resulted in tumor growth in 2 of 4 mice which received SAHA administration. In SAHA-treated xenografts, injection with cell numbers greater than 2,000 was capable of developing tumors in all experimental animals. Taken together, these findings indicate that SAHA might have limited effect on eradication of ALDH^br^ lung CSCs; moreover, the residual ALDH^br^ cells exhibited enhanced tumorigenic potential *in vivo*.

## DISCUSSION

In the current study, we used cells to develop a simpler method for isolating CSCs directly from excised H1299 xenografts, without any *in vitro* culture step. ALDH^br^ and Ds-Red double positive cells were regarded as putative CSCs, because the Ds-Red expression excluded other stem cell derived from the mice. By using ALDEFLUOR assay for detecting ALDH expression followed by standard FACS analysis, we successfully isolated ALDH^br^ cells and found that SAHA can suppress the growth of tumor xenografts and decreases the lung CSC population *in vivo.* Surprisingly, we found that residual ALDH^br^ cells from SAHA-treated mice exhibited properties of cancer stem cells, including high capacity of tumorigenesis, metastasis and stem cell-related gene expression.

CSCs and normal stem cells express similar surface markers, by which they can be identified and characterized. There has been no specific marker for the isolation and characterization of CSCs from different type of tumors to date [30]. The rigorous identification and isolation of tissue-specific CSCs has thus far been accomplished in only a few human organ systems. An overview of the techniques for the characterization of CSCs from the literatures shows controversial results. A Hoechst 33342 efflux assay in combination with flow cytometry has been used to identify and isolate “side populations,” which are enriched in cancer stem-like cells. Although the assay shows high efficiency in isolating side populations, the toxicity associated with the Hoechst 33342 dye creates bias by selectively injuring the non-side population cells that might affect subsequent functional stem cell assays *in vitro* and *in vivo* [31]. Furthermore, recent report even suggested that side populations in some tumors might not represent CSCs [32]. Flow cytometry methods using cell surface markers have been successfully applied to the mouse and human samples to isolate stem cell populations, but the choice of markers can greatly vary depending on different tissues or species. Besides, the markers used in mouse to sort specific stem cell populations are rarely valid in human. Non-SP cells or CD133^−/low^ cells can also generate tumors in NOD/SCID mice [4, 5]. These studies imply that only using one kind of technique such as cell surface markers or SP is not precise enough to isolate CSCs. Moreover, recent study further indicated that non-CS/PCs and CS/PCs of each subpopulation are interconvertible [33], which means that it is extremely difficult to identify CSCs in a tumor. Seeking a reliable marker to screen tumor-initiating cells without the influence on their biological characteristics becomes extremely critical.

We choose the ALDH marker as a starting point based on the prior work on lung CSCs, of which the cells with high ALDH activity were identified as putative CSCs [7]. Different from other CSC markers, ALDH^br^ cells are viable and are selectable by their fluorescence, thus avoiding the toxicity associated with Hoechst 33342 on the isolated cells. Furthermore, the ALDEFLUOR assay is based on the enzymatic activity of ALDH rather than cell surface phenotype and therefore it is not necessary to identify the specific cell surface markers that discriminate stem cells from their differentiated progeny of which the expression may also be dynamic and influenced by the local microenvironment. The majority of published reports on human lung cancer stem cells used commercially available lung cancer cell lines or “the cells” isolated from *in vitro* formed spheres [7, 34]. However, the biologic chracteristics of tumorspheres was far from CSCs in real tumors due to the distinct environments, a network of cytokines and growth factors, including the interaction with the mesenchymal stem cells, tumor associated fibroblasts, adipocytes, immune cells, and endothelial cells [9] which are lacking in the serum-free suspension culture system. Direct isolation of CSCs from xenograft models also has its defect. Macrophages, fibroblasts and other cells may express some stem cell markers, such as CD44 and CD24. As these cells are substantial populations in the tumor mass and may promote tumorigenesis, it is important to avoid their contamination in the lung cancer stem cell compartments. Unlike the previously described CSC phenotype, which might require the use of a combination of several surface antigens, our reporter gene-labelled cells and the ALDEFLUOR assay would be a more effective approach for defining and isolating an enriched lung CSC population from xenografts and might be potentially amenable to clinical applications.

Histone deacetylase inhibitors (HDACIs) have been shown to be promising drugs for the treatment of a number of hematological malignancies including cutaneous T-cell lymphoma [12] and peripheral T-cell in clinical trials [35]. However, therapeutic trials with the HDACIs in solid tumors have been disappointing, partly due to resistance to HDACIs themselves. HDACIs, including TSA and SAHA, have been applied to facilitate the development of embryo receiving somatic cell nuclear transfer and to induce ectopic expression of pluripotency factors for pluripotent stem cell derivation [36]. It implies a relationship between HDACIs and CSCs. A recent study has shown that SAHA could induce EMT phenotype and acquire cancer stem cell characteristics, which have been known to contribute to drug-resistant, cancer relapse and metastasis [19]. In the current study, we found up-regulation of the expression of metastatic genes in sorted ALDH^br^ cells treated with SAHA; this is in consistent with the increased CSC properties of SAHA treated cells. How does SAHA modulate CSCs in our xenograft model warrants further investigations.

The current study provided evidences of the effect of SAHA on lung CSCs. First, SAHA treatment significantly enhanced the stemness properties of H1299 lung cancer cells, of which the ALDH-expressing cells expressed high levels of stem cell-associated genes, efficiently formed tumorspheres and displayed high metastatic ability after SAHA administration. In contrast, previous studies revealed that SAHA could suppress the growth of tumor xenografts *in vivo* [37, 38]. The current study demonstrated that administration of SAHA can inhibit tumor growth and decrease ALDH^br^ population simultaneously in mice bearing human H1299 lung tumors. These findings are in consistent with the results of clinical trials that SAHA showed anticancer activity in patients with thyroid, renal cell and urothelial carcinomas [39], and may imply the possibility of SAHA against CSCs. However, the controversial results from *in vitro* and *in vivo* or clinical studies point out the importance of tumor microenvironment that defines the stem cell niche which might be affected by SAHA administration as well. Second, the residual ALDH^br^ cells from SAHA-treated mice exhibited more aggressive phenotype. Using SAHA-treated xenograft to derive the ALDH^br^ cells, we disclosed that these cells had much greater tumorigenicity and more enhanced invasiveness than that of tumor-bearing mice receiving vehicle alone. Third, the qRT-PCR results showed that ALDH^br^ cells markedly increase the expression of the genes associated with stemness, metastasis and drug resistance following SAHA treatment. Interpreting the above data based on the framework of the CSC theory implies that SAHA might worse the patient outcome by enhancing malignant characteristics of the residual CSCs and might be used to explain the limited effects of SAHA in clinical use.

SAHA has been the first HDACI approved by FDA and has been tested in several clinical trials. In clinical studies the effect of single use of SAHA seems to be minor. Our results disclosed the potential of SAHA to induce resistance to cancer therapy. The microarray gene expression data showed up-regulation of certain genes in SAHA-treated ALDH^br^ cells which are linked to high migration capacity, drug resistance, poor prognosis and could be a potential target for cancer therapy. The current study disclosed that MORF4L2, KCNMA1 and ASPM were up-regulated by SAHA treatment. MORF4L2 is a gene involving in drug metabolism, endocrine system development and function. MORF4L2, a component of the NuA4 histone acetyltransferase complex, involves the activation of oncogene and proto-oncogene-mediated growth induction, and replicative senescence; these result in higher levels of DNA repair and suppressed apoptosis [40]. KCNMA1 gene is related to cancer invasion and metastasis [20]. A previous study evaluated the relationship between ASPM and CSCs and revealed that the expression of ASPM correlates with tumor grade and increases at recurrence. ASPM was also considered as a marker for distinguishing stem-like cells from gliomas stromal cells [21]. Unexpectedly, we found the inconsistent results on the expression of ASPM and RASFF8 obtained from microarray and qPCR, respectively. Poor annotation or crosshybridization are not the reason for this inconsistency, because this experiment is repeated at least three times. Instead, it may be explained by a possible difference in splice variants detected by the two technologies, ASPM and RASFF8, of which more than one transcript were associated [41].

Although our results showed that a subpopulation of lung cancer cells exists within the H1299 xenografted tumors with markedly enhanced tumorigenic potential and stem cell properties after SAHA treatment, there are limitations to our study. First, high ALDH activity might not be as a universal marker for stem cells that may infer the use of ALDEFLUOR assay for identifying CSCs. It has also been demonstrated that isolated ALDH^br^ cells expressed lower proliferative rate and migration ability as compared with ALDH^lo^ cells in adipose tissue [42]. Thus, it is critical to carefully characterize the cells isolated based on high ALDH activity. Second, an established xenografted tumor rather than primary tumor directly obtained from the patient was investigated in this study. Further research using primary tumor is necessary to confirm the findings of this study. Third, the SAHA-induced CSCs activation was observed in H1299 cells, we will perform the parallel studies in other type of lung cancer cells to demonstrate that this effect globally exists in most of lung carcinoma cell lines.

We have successfully isolated and characterized CSCs *in vivo* from the xenografted H1299 lung cancers in mice rather than from the tumorspheres by conventional *in vitro* methods. The results of the current study revealed that SAHA treatment can suppress the tumor growth of H1299 lung cancer, decrease ALDH^br^ population but promote CSC characteristics, such as enhancement of the tumor initiating capacity and the expression of KCNMA1, MORF4L2 and ASPM genes in the residual subpopulation of cells with high ALDH activity residing in SAHA-treated xenografted tumor. We thus may propose that clinical use of SAHA alone in the treatment of solid tumor should proceed with a great caution.

## MATERIALS AND METHODS

### Cell lines

The H1299 human non–small cell lung cancer cell line was obtained from American Type Culture Collection (Rockville, MD) and maintained in RPMI 1640 (Gibco Laboratories, Grand Island, NY) supplemented with 10% fetal bovine serum (FBS), 2 mmol/L L-glutamine,100 μg/mL Streptomycin, and 100 units/mL Penicillin. All cells were maintained in a 37°C humidified 5% CO_2_ incubator. All media and cell culture reagents were purchased from Life Technologies (San Giuliano Milanese, Italy). Stable clones are generated by transfection of plasmids (Supplement 1.) and selected by appropriate antibiotics.

### SAHA preparation and administration

Suberoylanilide hydroxamic acid (SAHA) was synthesized as described previously [43]. Male BALB/c nude (nu/nu) mice with palpable H1299 subcutaneously xenografted tumors were randomly divided into two groups (at least four mice/group). All mice in each groups had tumors size (~100mm^3^) at the beginning of treatment. SAHA was dissolved in DMSO in a series of concentrations. Each mice daily received 100 mg/kg of SAHA by intraperitoneal (i.p.) administration for 10 days. The control group only received vehicle (DMSO). The animals were sacrificed on day 11 after SAHA treatment and the tumors were excised for further experiments.

### Tumor cell isolation

H1299 xenografts were sliced with a surgical blade and single-cell suspensions were generated by addition of 1 mg/ml (235 U/ml) collagenase I (Sigma-Aldrich, St. Louis, MO) for 60 min at 37°C with intermittent vortexing, followed by homogenization with cell dissociator (gentleMACS™, Miltenyi Biotec, Bergisch Gladbach, Germany), and sequentially passage through a 70 μm filter (Fisher Scientific, Pittsburgh, PA). Red blood cells were lysed using 1X Red Blood Cell Lysis Buffer (eBioscience, San Diego, CA). Cells were washed twice and subjected to FACS.

### ALDEFLUOR assay and separation of the ALDH^br^ population by FACS

To characterize the cells with high levels of ALDH (ALDH^br^), the ALDEFLUOR kit (Stem Cell Technologies, Vancouver, Canada) was utilized to isolate them with from tumor cells. A specific ALDH1 inhibitor, diethylaminobenzaldehyde (DEAB), was used as a negative control, and cells were analyzed with a FACSAria cell sorter (BD Biosciences, San Jose, CA). The sorting gates were established using the DsRed-expression cells for viability and the ALDEFLUOR-stained cells treated with DEAB as negative controls.

### Cell invasion assay

Cell invasion assay was performed following the previous literature with a Boyden chamber (8-μm pore size) [44].

### Sphere formation assay

The assay was performed following the protocol in previous literature [6]. Briefly, tumor cells or electronically sorted tumor cell subsets (5 × 10^4^ cells/mL) were seeded in ultralow attachment plates (Corning, USA) and grown in defined serum-free medium composed of RPMI 1640, hormone mixture B27 (Gibco, USA), 20 ng/mL EGF (epidermal growth factor; Sigma-Aldrich, USA), 10 ng/mL bFGF (basic fibroblast growth factor; Invitrogen, USA) and 1 μg/mL heparin. The number of spheroids was counted after 14 days later.

### Quantitative real-time PCR

To determine the changes in the expression level of ALDH^br^ cells in microarray data, we performed Quantitative Real-Time RT-PCR. The total mRNA was extracted using Direct-zol RNA MiniPrep kit with on column DNA digestion (Zymo Research Corp., California, USA). The first strand of cDNA was generated from 1 μg total RNA using oligo-dT primer and SuperScript® III Reverse Transcriprase (Invitrogen). Quantitative real-time PCR was run on StepOnePlus real-time PCR system (Applied Biosystems, Carlsbad, USA) with the preset PCR program, using TaqMan primers for β-actin, ASPM, KCNMA1, RASSF8, MORF4L2 and TaqMan Universal PCR Master Mix (Applied Biosystems, Carlsbad, USA) for dection. The cycle number when the fluorescence first reachs a preset threshold (Ct), allows the quantification of the specific template concentraction. Transcripts of the housekeeping gene β-actin in the same incubations were used for internal normalization.

### *In vivo* tumor formation assays

All the animal protocols in this study were in accordance with the Animal Care and Use Committee of our institution. BALB/C nude mice were used to assess the *in vivo* stem cell properties of the ALDEFLUOR-positive population with SAHA treatment, compared to the same population with administration of vehicle only (DMSO). The H1299 xenografted cells were sorted as previously described and the tumorigenicity of each populations of H1299 cells with or without SAHA treatment was tested by inoculation of limiting dilutions of cells (1,000, 2,000, 4,000, and 8,000 cells) mixed with Matrigel (BD Biosciences, Mississauga, Canada; 1:1) into the anaesthetized 6-week-old nude mice, subcutaneously. Tumor size was measured weekly using a caliper and tumor volume was calculated using the following formula: tumor volume = length × width × thickness × 0.523.

### Microarray analysis

The Affymetrix Human U133 plus 2.0 whole genome array was used in this study. Total RNA extraction, cRNA probe preparation, array hybridization, feature selection and computational analysis were performed as previously described [45]. The *q*-value was calculated to control the multiple testing errors in differential expression analysis, as previously described [45]. The heat map was created by the dChip software. More than 2 fold changes in all chip data were considered as up-regulated.

### Immunofluorescence-microscopy staining

Immunofluorescence staining was performed using antibodies for ALDH1A1, CD133 and vimentin as previously described [46]. The characteristics of the antibodies used are listed in Supplementary Information, Table S1.

## SUPPLEMENTARY MATERIALS, TABLE AND FIGURES



## References

[1] Boman BM, Wicha MS (2008). Cancer stem cells: a step toward the cure. J Clin Oncol.

[2] Pajonk F, Vlashi E, McBride WH (2010). Radiation resistance of cancer stem cells: the 4 R's of radiobiology revisited. Stem cells.

[3] Cao L, Zhou Y, Zhai B, Liao J, Xu W, Zhang R, Li J, Zhang Y, Chen L, Qian H (2011). Sphere-forming cell subpopulations with cancer stem cell properties in human hepatoma cell lines. BMC Gastroenterol.

[4] Xu W, Lin H, Zhang Y, Chen X, Hua B, Hou W, Qi X, Pei Y, Zhu X, Zhao Z (2011). Compound Kushen Injection suppresses human breast cancer stem-like cells by down-regulating the canonical Wnt/b-catenin pathway. J Exp Clin Cancer Res.

[5] Friedman S, Lu M, Schultz A, Thomas D, Lin R-Y (2009). CD133+ anaplastic thyroid cancer cells initiate tumors in immunodeficient mice and are regulated by thyrotropin. PLoS One.

[6] Ginestier C, Hur MH, Charafe-Jauffret E, Monville F, Dutcher J, Brown M, Jacquemier J, Viens P, Kleer CG, Liu S, Schott A, Hayes D, Birnbaum D, Wicha MS, Dontu G (2007). ALDH1 is a marker of normal and malignant human mammary stem cells and a predictor of poor clinical outcome. Cell Stem Cell.

[7] Sullivan JP, Spinola M, Dodge M, Raso MG, Behrens C, Gao B, Schuster K, Shao C, Larsen JE, Sullivan LA (2010). Aldehyde dehydrogenase activity selects for lung adenocarcinoma stem cells dependent on notch signaling. Cancer Res.

[8] Ma S, Chan KW, Lee TK, Tang KH, Wo JY, Zheng BJ, Guan XY (2008). Aldehyde dehydrogenase discriminates the CD133 liver cancer stem cell populations. Molecular cancer research: MCR.

[9] Huang EH, Hynes MJ, Zhang T, Ginestier C, Dontu G, Appelman H, Fields JZ, Wicha MS, Boman BM (2009). Aldehyde dehydrogenase 1 is a marker for normal and malignant human colonic stem cells (SC) and tracks SC overpopulation during colon tumorigenesis. Cancer Res.

[10] David G, Alland L, Hong S-H, Wong C-W, DePinho RA, Dejean A (1998). Histone deacetylase associated with mSin3A mediates repression by the acute promyelocytic leukemia-associated PLZF protein. Oncogene.

[11] Richon VM, Sandhoff TW, Rifkind RA, Marks PA (2000). Histone deacetylase inhibitor selectively induces p21WAF1 expression and gene-associated histone acetylation. Proc Natl Acad Sci USA.

[12] Duvic M, Vu J (2007). Vorinostat: a new oral histone deacetylase inhibitor approved for cutaneous T-cell lymphoma. Expert Opin Investig Drugs.

[13] Orzan F, Pellegatta S, Poliani P, Pisati F, Caldera V, Menghi F, Kapetis D, Marras C, Schiffer D, Finocchiaro G (2011). Enhancer of Zeste 2 (EZH2) is up-regulated in malignant gliomas and in glioma stem-like cells. Neuropathol Appl Neurobiol.

[14] Nalls D, Tang SN, Rodova M, Srivastava RK, Shankar S (2011). Targeting epigenetic regulation of miR-34a for treatment of pancreatic cancer by inhibition of pancreatic cancer stem cells. PLoS One.

[15] Kong D, Ahmad A, Bao B, Li Y, Banerjee S, Sarkar FH (2012). Histone deacetylase inhibitors induce epithelial-to-mesenchymal transition in prostate cancer cells. PLoS One.

[16] Jiang G-M, Wang H-S, Zhang F, Zhang K-S, Liu Z-C, Fang R, Wang H, Cai S-H, Du J (2012). Histone deacetylase inhibitors induction of epithelial-mesenchymal transitions via up-regulation of Snail facilitates cancer progression. Biochimica et biophysica acta.

[17] Kong D, Banerjee S, Ahmad A, Li Y, Wang Z, Sethi S, Sarkar FH (2010). Epithelial to mesenchymal transition is mechanistically linked with stem cell signatures in prostate cancer cells. PLoS One.

[18] Kong D, Li Y, Wang Z, Sarkar FH (2011). Cancer stem cells and epithelial-to-mesenchymal transition (EMT)-phenotypic cells: are they cousins or twins?. Cancers.

[19] Mani SA, Guo W, Liao MJ, Eaton EN, Ayyanan A, Zhou AY, Brooks M, Reinhard F, Zhang CC, Shipitsin M, Campbell LL, Polyak K, Brisken C, Yang J, Weinberg RA (2008). The epithelial-mesenchymal transition generates cells with properties of stem cells. Cell.

[20] Khaitan D, Sankpal UT, Weksler B, Meister EA, Romero IA, Couraud P-O, Ningaraj NS (2009). Role of KCNMA1 gene in breast cancer invasion and metastasis to brain. BMC cancer.

[21] Bikeye S-NN, Colin C, Marie Y, Vampouille R, Ravassard P, Rousseau A, Boisselier B, Idbaih A, Calvo CF, Leuraud P (2010). ASPM-associated stem cell proliferation is involved in malignant progression of gliomas and constitutes an attractive therapeutic target. Cancer cell international.

[22] Serrano D, Bleau AM, Fernandez-Garcia I, Fernandez-Marcelo T, Iniesta P, Ortiz-de-Solorzano C, Calvo A (2011). Inhibition of telomerase activity preferentially targets aldehyde dehydrogenase-positive cancer stem-like cells in lung cancer. Molecular cancer.

[23] Hahn SA, Seymour AB, Hoque AS, Schutte M, da Costa LT, Redston MS, Caldas C, Weinstein CL, Fischer A, Yeo CJ (1995). Allelotype of pancreatic adenocarcinoma using xenograft enrichment. Cancer Res.

[24] Al-Hajj M, Wicha MS, Benito-Hernandez A, Morrison SJ, Clarke MF (2003). Prospective identification of tumorigenic breast cancer cells. Proc Natl Acad Sci USA.

[25] Hemmati HD, Nakano I, Lazareff JA, Masterman-Smith M, Geschwind DH, Bronner-Fraser M, Kornblum HI (2003). Cancerous stem cells can arise from pediatric brain tumors. Proc Natl Acad Sci USA.

[26] Lapidot T, Sirard C, Vormoor J, Murdoch B, Hoang T, Caceres-Cortes J, Minden M, Paterson B, Caligiuri MA, Dick JE (1994). A cell initiating human acute myeloid leukaemia after transplantation into SCID mice.

[27] Li C, Heidt DG, Dalerba P, Burant CF, Zhang L, Adsay V, Wicha M, Clarke MF, Simeone DM (2007). Identification of pancreatic cancer stem cells. Cancer Res.

[28] O'Brien CA, Pollett A, Gallinger S, Dick JE (2006). A human colon cancer cell capable of initiating tumour growth in immunodeficient mice. Nature.

[29] Schatton T, Murphy GF, Frank NY, Yamaura K, Waaga-Gasser AM, Gasser M, Zhan Q, Jordan S, Duncan LM, Weishaupt C (2008). Identification of cells initiating human melanomas. Nature.

[30] Visvader JE, Lindeman GJ (2008). Cancer stem cells in solid tumours: accumulating evidence and unresolved questions. Nature Reviews Cancer.

[31] Wu A, Oh S, Wiesner SM, Ericson K, Chen L, Hall WA, Champoux PE, Low WC, Ohlfest JR (2008). Persistence of CD133+ cells in human and mouse glioma cell lines: detailed characterization of GL261 glioma cells with cancer stem cell-like properties. Stem cells and development.

[32] Burkert J, Otto WR, Wright NA (2008). Side populations of gastrointestinal cancers are not enriched in stem cells. The Journal of pathology.

[33] Akunuru S, James Zhai Q, Zheng Y (2012). Non-small cell lung cancer stem/progenitor cells are enriched in multiple distinct phenotypic subpopulations and exhibit plasticity. Cell Death Dis.

[34] Jiang F, Qiu Q, Khanna A, Todd NW, Deepak J, Xing L, Wang H, Liu Z, Su Y, Stass SA, Katz RL (2009). Aldehyde dehydrogenase 1 is a tumor stem cell-associated marker in lung cancer. Molecular cancer research: MCR.

[35] Piekarz RL, Frye R, Prince HM, Kirschbaum MH, Zain J, Allen SL, Jaffe ES, Ling A, Turner M, Peer CJ, Figg WD, Steinberg SM, Smith S, Joske D, Lewis I, Hutchins L (2011). Phase 2 trial of romidepsin in patients with peripheral T-cell lymphoma. Blood.

[36] Huangfu D, Maehr R, Guo W, Eijkelenboom A, Snitow M, Chen AE, Melton DA (2008). Induction of pluripotent stem cells by defined factors is greatly improved by small-molecule compounds. Nature biotechnology.

[37] Butler LM, Agus DB, Scher HI, Higgins B, Rose A, Cordon-Cardo C, Thaler HT, Rifkind RA, Marks PA, Richon VM (2000). Suberoylanilide hydroxamic acid, an inhibitor of histone deacetylase, suppresses the growth of prostate cancer cells *in vitro* and *in vivo*. Cancer Res.

[38] Lee YJ, Won AJ, Lee J, Jung JH, Yoon S, Lee BM, Kim HS (2012). Molecular mechanism of SAHA on regulation of autophagic cell death in tamoxifen-resistant MCF-7 breast cancer cells. International journal of medical sciences.

[39] Kelly WK, O'Connor OA, Krug LM, Chiao JH, Heaney M, Curley T, MacGregore-Cortelli B, Tong W, Secrist JP, Schwartz L (2005). Phase I study of an oral histone deacetylase inhibitor, suberoylanilide hydroxamic acid, in patients with advanced cancer. J Clin Oncol.

[40] Kuete V, Eichhorn T, Wiench B, Krusche B, Efferth T (2012). Cytotoxicity, anti-angiogenic, apoptotic effects and transcript profiling of a naturally occurring naphthyl butenone, guieranone A. Cell division.

[41] Kersey PJ, Allen JE, Christensen M, Davis P, Falin LJ, Grabmueller C, Hughes DS, Humphrey J, Kerhornou A, Khobova J, Langridge N, McDowall MD, Maheswari U, Maslen G, Nuhn M, Ong CK (2014). Ensembl Genomes 2013: scaling up access to genome-wide data. Nucleic Acids Res.

[42] Wang L, Park P, Zhang H, La Marca F, Lin CY (2011). Prospective identification of tumorigenic osteosarcoma cancer stem cells in OS99-1 cells based on high aldehyde dehydrogenase activity. International journal of cancer Journal international du cancer.

[43] Richon V, Webb Y, Merger R, Sheppard T, Jursic B, Ngo L, Civoli F, Breslow R, Rifkind R, Marks P (1996). Second generation hybrid polar compounds are potent inducers of transformed cell differentiation. Proc Natl Acad Sci USA.

[44] Yang MH, Hsu DS, Wang HW, Wang HJ, Lan HY, Yang WH, Huang CH, Kao SY, Tzeng CH, Tai SK, Chang SY, Lee OK, Wu KJ (2010). Bmi1 is essential in Twist1-induced epithelial-mesenchymal transition. Nat Cell Biol.

[45] Wang H-W, Trotter MW, Lagos D, Bourboulia D, Henderson S, Mäkinen T, Elliman S, Flanagan AM, Alitalo K, Boshoff C (2004). Kaposi sarcoma herpesvirus–induced cellular reprogramming contributes to the lymphatic endothelial gene expression in Kaposi sarcoma. Nature genetics.

[46] Yang M, Chang S, Chiou S, Liu C, Chi C, Chen P, Teng S, Wu K (2006). Overexpression of NBS1 induces epithelial–mesenchymal transition and co-expression of NBS1 and Snail predicts metastasis of head and neck cancer. Oncogene.

