# Supplementary material for: Enhancement of tumor initiation and expression of KCNMA1, MORF4L2 and ASPM genes in the adenocarcinoma of lung xenograft after vorinostat treatment
Source: Oncotarget. 2015 Mar 12;6(11):8663–75. doi: 10.18632/oncotarget.3536 (PMC4496174; doi:10.18632/oncotarget.3536)
Supplement: Supplementary file 1 [file oncotarget-06-8663-s001.pdf]

Enhancement of tumor initiation and expression of KCNMA1, MORF4L2 and ASPM genes in the adenocarcinoma of lung xenograft after vorinostat treatment

Supplementary Material

Table S1: List of proteins tested by antibodies and characteristics of the corresponding antibodies

Protein	Assay	Antibody	Origin	Dilution	Incubation period
CD133	IF	#130-090-422, Miltenyi Biotec Inc.	mmab	1/100	overnight, 4°C
ALDH1A1	IF	Ab51028, Abcam plc.	rpab	1/100	overnight, 4°C
Vimentin	IF	V6630, Sigma	mmab	1/100	overnight, 4°C

Abbreviations: IF, immunofluorescence; mmab, mouse monoclonal antibody; rpab, rabbit polyclonal antibody

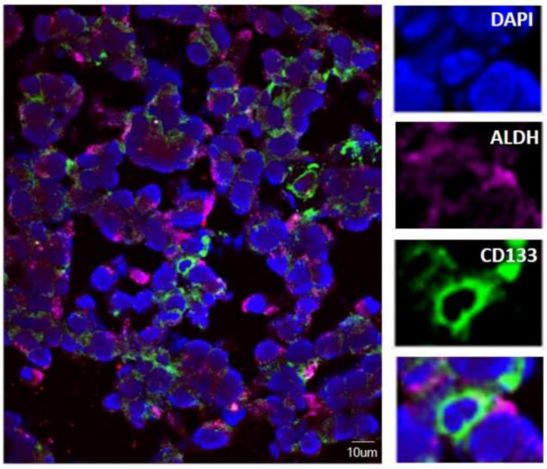

Supplement Figure 1: Construction of triple fusion reporter gene

Briefly, the monomeric DsRed expression plasmid, pDsRed-Monomer-C1, driven by CMV enhancer/pormoter is purchased from Clontech (BD science. Inc. USA). The pDsRed-Monomer-C1 digested with restriction enzyme of BamH I and Xba I (New England Biolabs Inc., USA) is purified from 1% agarose gel by PCR/Gel Extraction kit (Geneaid Inc., Taiwan) and used as cloning vector. The truncated tksr39 gene is amplified from the plasmid pttksr39 (generous gifts from Professor FD Chen, TransWorld University, Taiwan) by polymerase chain reaction (PCR) with 5'-end primer ttkUp/BamHI (5'-CAA GAC GGA TCC TCT GGT AAA ATG CCC ACG CTA CTG C-3') , 3'-end primer ttkDn-XbaI (5'-GTA TTC TCT AGA TCA GTT AGC CTC CCC CAT C-3') and the proof-reading KOD Taq DNA polymerase (Novagen Inc., USA). The ttkSR39 PCR products are purified and subjected to restriction enzyme of BamH I and Xba I (New England Biolabs Inc., USA) digestion and

then the purified ttksr39 insert DNAs are ligated with BamHI-XbaI digested pDsRed-Monomer-C1 vector by T4 DNA ligase (New England Biolabs Inc., USA) generating a DsRedm-ttksr39 dual fusion reporter genetic construct. The DsRedm-ttksr39 plasmid is digested with restriction enzyme of EcoR I and Sal I (New England Biolabs Inc., USA) and used for a cloning vector. The fl gene from the pGL3 basic plasmid (Promega Corporation, Madison, Wi, USA.) is amplified by PCR using the same 5'-end primer FLucUp-EcoR I 5'-AGC ATC GAA TTC TGA GGA CGC CAA AAA CAT AAA G-3', the 3'end primer FlucDn-Sal I 5'-CTA GTA GTC GAC AGC AAT CTT TCC GCC CTT CT-3' and the proof-reading KOD Taq DNA polymerase (Novagen Inc., USA). After purification, the fl PCR products are digested with restriction enzyme of EcoR I and Sal I and then are ligated with the EcoR I-Sal I digested DsRedm-ttksr39 cloning vector to create the DsRedm-fl-ttksr39 triple fusion reporter genetic construct. The DNA coding sequences of all constructs were verified by DNA sequencing service (MISSON BIOTECH Inc., Taiwan) using ABI model 3730 DNA sequencer.

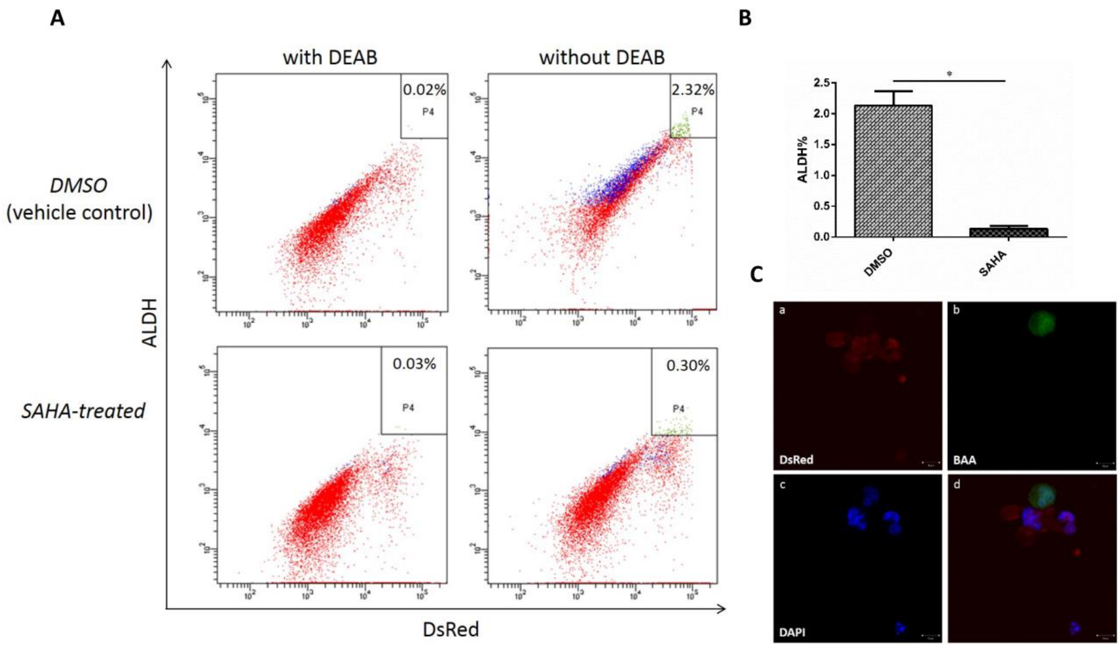

Supplement Figure 2: Decreased number of ALDH^{br} cells shown in xenografts treated with SAHA. (A) Representative flow cytometric analysis of ALDH^{br} cells in H1299 xenografts; percentage of ALDH^{br} cells was 0.20±0.04% in SAHA-treated xenograft tumors (bottom). Control samples incubated with the inhibitor, DEAB, were used to ensure the identification of ALDH^{br} and ALDH^{lo} cells. Data represent three independent experiments (mean ± SD). (B) Percentage of ALDH^{br} cells in SAHA-treated H1299 xenografts had around 11-fold reduction compared with ALDH^{br} cells isolated from vehicle-treated controls. (C) ALDH^{br} cells can be observed by confocal microscopy through accumulation of BAA (green, arrow) with nuclei identified by DAPI (blue).
